# Adherence to dietary recommendations by socioeconomic status in the United Kingdom biobank cohort study

**DOI:** 10.3389/fnut.2024.1349538

**Published:** 2024-05-01

**Authors:** Fernanda Carrasco-Marín, Solange Parra-Soto, Jirapitcha Bonpoor, Nathan Phillips, Atefeh Talebi, Fanny Petermann-Rocha, Jill Pell, Frederick Ho, Nicolás Martínez-Maturana, Carlos Celis-Morales, Rafael Molina-Luque, Guillermo Molina-Recio

**Affiliations:** ^1^Departamento de Enfermería, Farmacología y Fisioterapia, Facultad de Medicina y Enfermería, Universidad de Córdoba, Córdoba, Spain; ^2^School of Cardiovascular and Metabolic Health, University of Glasgow, Glasgow, United Kingdom; ^3^Centro de Vida Saludable, Universidad de Concepción, Concepción, Chile; ^4^Department of Nutrition and Public Health, Universidad del Bío-Bío, Chillán, Chile; ^5^Faculty of Public Health, Chalermphrakiat Sakon Nakhon Province Campus, Kasetsart University, Sakon Nakhon, Thailand; ^6^Centro de Investigación Biomédica, Facultad de Medicina, Universidad Diego Portales, Santiago, Chile; ^7^School of Health and Wellbeing, University of Glasgow, Glasgow, United Kingdom; ^8^Departamento de Ciencias Preclínicas, Facultad de Medicina, Universidad de La Frontera, Temuco, Chile; ^9^Human Performance Lab, Education, Physical Activity and Health Research Unit, University Católica del Maule, Talca, Chile; ^10^Grupo Asociado de Investigación Estilos de Vida, Innovación y Salud, Instituto Maimónides de Investigación Biomédica de Córdoba, Córdoba, Spain

**Keywords:** diet, socioeconomic status, education, income, deprivation

## Abstract

**Introduction:**

Understanding how socioeconomic markers interact could inform future policies aimed at increasing adherence to a healthy diet.

**Methods:**

This cross-sectional study included 437,860 participants from the UK Biobank. Dietary intake was self-reported. Were used as measures socioeconomic education level, income and Townsend deprivation index. A healthy diet score was defined using current dietary recommendations for nine food items and one point was assigned for meeting the recommendation for each. Good adherence to a healthy diet was defined as the top 75th percentile, while poor adherence was defined as the lowest 25th percentile. Poisson regression was used to investigate adherence to dietary recommendations.

**Results:**

There were significant trends whereby diet scores tended to be less healthy as deprivation markers increased. The diet score trends were greater for education compared to area deprivation and income. Compared to participants with the highest level of education, those with the lowest education were found to be 48% less likely to adhere to a healthy diet (95% Confidence Interval [CI]: 0.60–0.64). Additionally, participants with the lowest income level were 33% less likely to maintain a healthy diet (95% CI: 0.73–0.81), and those in the most deprived areas were 13% less likely (95% CI: 0.84–0.91).

**Discussion/conclussion:**

Among the three measured proxies of socioeconomic status – education, income, and area deprivation – low education emerged as the strongest factor associated with lower adherence to a healthy diet.

## 1 Introduction

Poor diet is a key predictor of non-communicable diseases and premature mortality (1, 2). The Global Burden of Diseases diet working group’s latest report estimated that poor dietary habits, characterized by inadequate intake of essential nutrients from oily fish, whole grains, nuts, fruits, and vegetables, coupled with excessive consumption of salt, red meat, processed meat, and sugary drinks, contribute to approximately 11 million deaths and 255 million disability-adjusted life-years globally (3). Intriguingly, the health burden from poor diet surpasses that of well-known risk factors such as physical inactivity and smoking (4).

The detrimental impact of unhealthy diets on health is markedly pronounced across various socioeconomic statuses (SESs). Evidence consistently shows that individuals in more deprived areas are less inclined to follow current dietary guidelines (5, 6). For example, a study in Italy demonstrated that higher adherence to a Mediterranean diet, which is linked to a lower risk of cardiovascular diseases (CVDs), was primarily observed in those with higher SES, in contrast to their lower SES counterparts (7, 8). Correspondingly, the 2008 East of England Lifestyle Survey involving 26,290 adults highlighted that residents of deprived neighborhoods were less likely to meet fruit and vegetable intake recommendations (9). While the links between individual-level socioeconomic factors, such as education and income, and dietary adherence have been extensively explored, the relationship between dietary habits and neighborhood-level deprivation is not as well understood.

Comprehending the representation of various resources and challenges by different socioeconomic markers is crucial. This understanding is vital to unravel how these markers influence adherence to healthy dietary recommendations (10, 11). Furthermore, there is a pressing need to identify which specific foods recommended in dietary guidelines are most susceptible to socioeconomic-patterned suboptimal intake or overconsumption (12). Such insights are essential for informing future public health policies and identifying new targets for both individual and population-level interventions. Therefore, the primary objective of this study is to examine the association between individual-level and area-level measures of SES and adherence to dietary recommendations within the UK Biobank cohort.

## 2 Materials and methods

Between 2006 and 2010, UK Biobank recruited more than 500,000 participants (5.5% response rate), aged 37 to 73 years from Scotland, Wales, and England. Participants attended one of the 22 assessment centers where they completed a touch-screen questionnaire, had physical measurements taken, and provided biological samples, as described in detail elsewhere^1^ (13).

### 2.1 Socioeconomic measurements

For the SES exposure, three measures were examined: Townsend score (area-level deprivation), annual household income (household-level), and maximum education attainment (individual-level). Townsend scores are derived from data on unemployment, car ownership, household overcrowding, and owner occupation aggregated at postcode area (14). Townsend scores were assigned to participants based on their address at recruitment and were calculated immediately prior to recruitment using data from the preceding national census data (2001). Higher Townsend scores equate to higher levels of socioeconomic deprivation. For the study, the deprivation index was categorized into quintiles. Household income (£/year) was self-reported at baseline and categorized as: < 18,000; 18,000–30,999; 31,000–51,999; 52,000–100,000; and > 100,000. Educational attainment, derived from self-reported qualifications at baseline and based on previous UK Biobank analyses using the International Standard Classification of Education (15), was categorized ordinally as college or university degree, A-levels/AS levels/equivalent (pre-university qualifications), O-levels/GCSEs/equivalent (qualifications taken prior to A or AS-level), CSEs/equivalent (qualifications typically taken at aged 16 years prior to A or AS-level, but aimed at less able pupils than those taking O-levels), and none of the above. NVQ/HND/HNC/equivalent (work-based vocational/higher educational qualifications) and “Other professional qualifications” were discounted as it is unclear where these would fit in the hierarchy.

### 2.2 Dietary exposures

To analyze diet, a cumulative dietary risk score by Petermann-Rocha (6) was used, for which the purposes of this study were inverted. A food frequency questionnaire was self-completed using a touch-screen at baseline to assess each participant’s diet. Nine food items previously included in the UK and European dietary guidelines (processed meat, red meat, total fish, milk, spread type, cereal intake, salt added to food, water, and fruits and vegetables) were included in this study. All food items were dichotomized into meeting or not meeting dietary recommendations using cutoffs derived from the UK and European food-based dietary intake guidelines where these existed (the Eatwell guide and the Food-Based Dietary Guidelines from the European Food Safety Authority) (16, 17). For those food items without a specific recommendation of intake (cereal intake), the median was used (18). More details are provided in Supplementary Table S1. To derive a diet score, one point was assigned to participants for each “healthy” recommendation defined as fruits and vegetables (>5 servings/day), processed meat (less than once per week), red meat (less than once/week), oily fish (more than twice per week), cereal (more than five bowls per week), water (more than six glasses per day), consumption of dairy products (semi-skimmed/skimmed), and adding salt to food as a proxy of salt intake (never/rarely spread intake). Points scored for each of these nine food items were then summed for each participant to derive an unweighted score with a minimum score of 0, which represented the most unhealthy diet, and a maximum score of 9, which represented the most healthy diet (6). The 9-point score was then categorized into quartiles according to their score range. Participants in the top 25th percentile, equivalent to a score of 7–9 points, were classified as the healthiest, while individuals in the lowest 25th percentile, equivalent to a score of 0–3 points, were classified as the least healthy. More details about the score validation can be found elsewhere (6).

### 2.3 Covariates

Age was calculated from the date of birth and the date of baseline assessment. Ethnicity was self-reported and categorized as Caucasian, South Asian, African descent, Chinese, and mixed or other ethnic backgrounds. Anthropometric measurements, including height and body weight, were taken by trained nurses during the initial assessment. Body mass index (BMI) was calculated as (weight in kg)/(height in m)^2^, and the WHO criteria were applied to categorize participants into underweight <18.5 kg.m^2^, normal weight 18.5–24.9 kg.m^2^, overweight 25.0–29.9 kg.m^2^, and obese ≥30.0 kg.m^2^ (19). Smoking status was categorized as a never, former, or current smoker. Medical history, including physician diagnosis of depression, stroke, angina, heart attack, hypertension, cancer, diabetes, hypertension, or other illnesses, was self-reported at the baseline assessment visit.

### 2.4 Ethics approval

The UK Biobank study was approved by the North West Multi-Centre Research Ethics Committee (NHS National Research Ethics Service 16/NW/0274). Participants provided written informed consent for data collection, data analysis, and record linkage. This study is part of UK Biobank project 7,155.

### 2.5 Statistical analyses

Descriptive characteristics by category of the diet score are presented as means with standard deviations (SDs) for quantitative variables or as frequency with percentage for categorical variables. Differences for continuous variables were assessed using a *t*-test and chi^2^ for categorical variables for the cohort characteristics presented in Table 1. The score means and their 95% confidence intervals (CIs) by categories of SES were derived using linear regression analysis. Trend analyses were conducted by fitting the score as an ordinal exposure into the model. These analyses were adjusted for age, sex, ethnicity, smoking, BMI, and multimorbidity.

**Table 1:** Cohort characteristics by high and low adherence to a healthy diet.

Diet score	Least healthy diet (≤3 points)		Most healthy diet (≥7 points)		*p*-value
*n*	94,000		31,569		
**Sociodemographics**					
Age, years, mean (SD)	54.9	(8.2)	56.7	(7.9)	<0.001
Sex, *n* (%)					<0.001
Female	39,250	(41.8)	21,583	(68.4)	
Male	54,750	(58.2)	9,986	(31.6)	
Deprivation, *n* (%)					<0.001
Lowest	17,515	(18.6)	6,876	(21.8)	
Lower/Middle	17,764	(18.9)	6,582	(20.9)	
Middle	18,461	(19.6)	6,561	(20.8)	
Higher/Middle	19,611	(20.9)	6,230	(19.7)	
Highest deprivation	20,649	(22.0)	5,320	(16.9)	
Education Qualifications, *n* (%)					<0.001
College or University degree	32,613	(43.1)	14,408	(52.7)	
A levels/AS levels or equivalent	12,178	(16.1)	4,281	(15.6)	
0 levels/GCSEs or equivalent	23,661	(31.3)	7,222	(26.4)	
SEs or equivalent /NVQ or HND or HNC	7,140	(9.5)	1,457	(5.3)	
Income, *n* (%)					<0.001
Less than 18.000	21,653	(23.0)	6,673	(21.1)	
18.000 to 30.999	22,383	(23.8)	8,007	(25.4)	
31.000 to 51.999	24,697	(26.3)	8,283	(26.2)	
52.000 to 100.000	20,004	(21.3)	6,595	(20.9)	
Greater than 100.000	5,263	(5.6)	2,011	(6.4)	
Ethnicity, *n* (%)					<0.001
Caucasian	89,740	(95.4)	30.268	(95.9)	
Mixed	621	(0.7)	147	(0.47)	
South Asian	1,359	(1.5)	481	(1.52)	
African descent	1,354	(1.4)	354	(1.12)	
Chinese	310	(0.3)	56	(0.18)	
any other	616	(0.7)	263	(0.83)	
**Nutritional status**					
BMI, mean(SD)	27.9	(4.86)	26.3	(4.48)	<0.001
BMI Categories, *n* (%)					<0.001
Underweight (<18.5 kg/m^2^)	463	(0.5)	223	(0.71)	
Normal (18.5–24.9 kg/m^2^)	26,590	(28.4)	13,472	(42.8)	
Overweight (25.0–29.9 kg/m^2^)	40,723	(43.5)	12,447	(39.6)	
Obese (≥30,0 kg/m^2^)	25,827	(27.6)	5,329	(16.9)	
**Lifestyle behaviors**					
Smoking status, *n* (%)					<0.001
Never/Previous	78,928	(84.0)	30,170	(95.6)	
Current	15,072	(16.0)	1,399	(4.4)	
**Prevalent diseases**					
Cardiovascular diseases, *n* (%)	26,670	(28,4)	8,567	(27,1)	<0.001
Hypertension, *n* (%)	4,460	(4.7)	1.246	(4.0)	<0.001
Diabetes, *n* (%)	23.752	(25.3)	7.564	(23.9)	<0.001

Data are presented as mean and standard deviation for continuous variables and as frequency and percentage for categorical variables. %TE, percentage of total energy intake; BMI, body mass index. Differences for continuous variables were tested using a *t*-test and chi^2^ for categorical variables.

High adherence to a healthy diet score was defined as individuals within the top 75th percentile of the healthy diet score (7–9 points), while low adherence was defined as the bottom 25th percentile of the diet score (0–3 points). Poisson regression analyses were conducted to investigate adherence to a healthy diet by categories of SES. Poisson regression was used instead of logistic regression to correct the association estimates when the outcome is common (>10%). Those participants in the most affluent, most educated, or least deprived categories were used as the reference group. Results were reported as risk ratios and their 95% CI. Analyses were adjusted for age, sex, ethnicity, BMI, smoking, and multimorbidity. Similar analyses were performed to investigate the association between combined socioeconomic markers and adherence to a healthy diet. The reference group was those with the most advantaged SES (i.e., highest education and lowest deprivation or higher income). A risk matrix was derived using risk ratio estimates adjusted for age, sex, ethnicity, BMI, smoking, and multimorbidity. The association between food intake according to dietary recommendations, expressed as mean, and categories of SES were obtained using linear regression analysis. The results were presented as regression coefficients with their respective 95% CIs. Trend *p*-values were estimated using linear regression analysis. All analyses were conducted using the software STATA 15 (StataCorp, College Station, TX).

## 3 Results

Out of the 501,897 individuals enrolled in the UK Biobank study, 437,860 participants (87.2%) had complete data on diet, socioeconomic markers, and other relevant covariates and were thus included in this analysis. Table 1 presents the main characteristics of the cohort, categorized by tertiles of adherence to healthy dietary recommendations. In summary, compared to those with high adherence to a healthy diet, the group with low adherence included a higher percentage of younger participants, predominantly men, who were more likely to be obese and current smokers. Additionally, this group generally had lower educational qualifications and a marginally higher prevalence of comorbidities.

**Figure 1:**
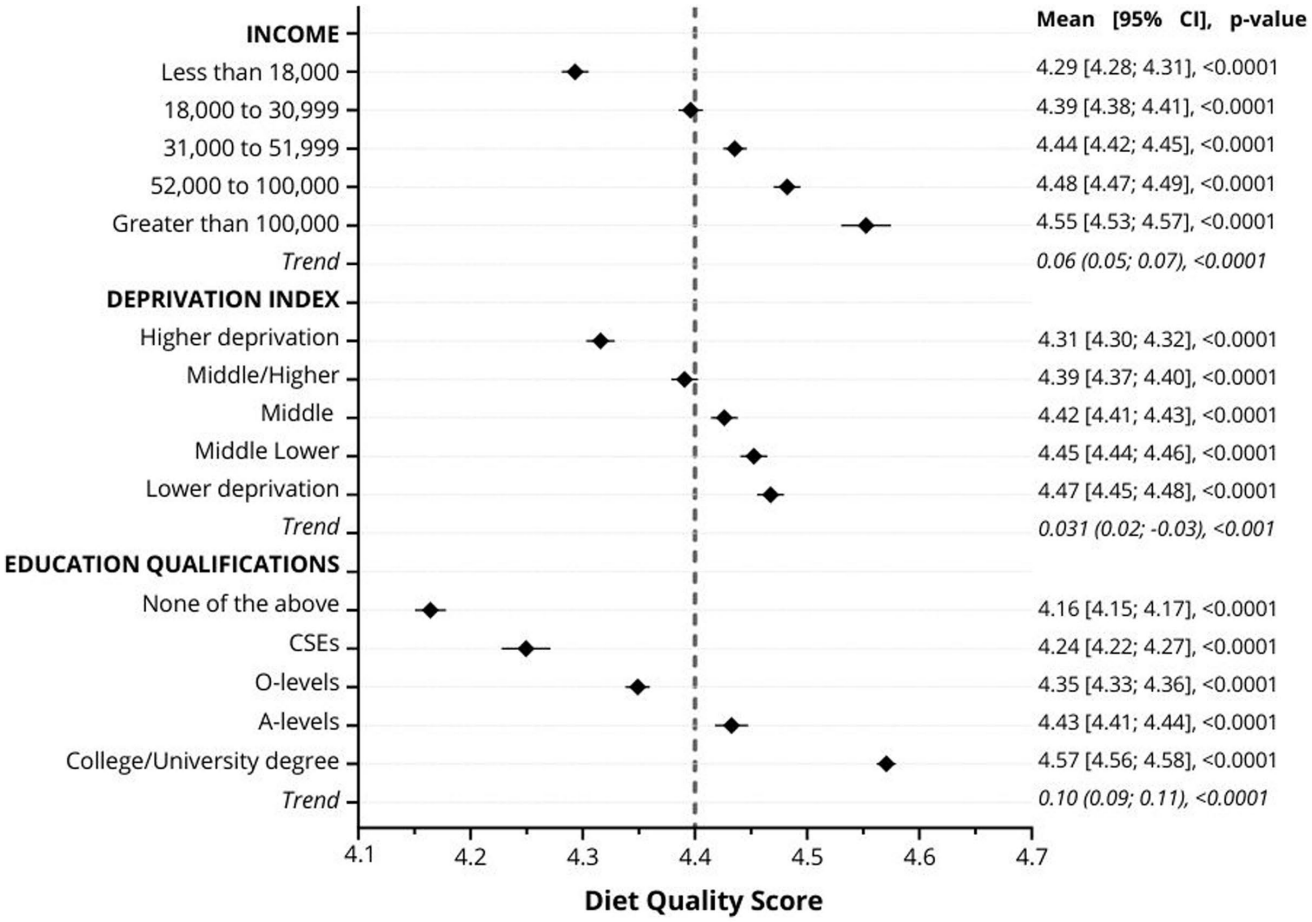
Mean diet quality score by categories of education, income, and deprivation. Data are presented as the mean of the diet quality score and their 95%CI. Analyses were adjusted for age, sex, ethnicity, smoking, multimorbidity, and BMI. The dotted line represents the population median. The trend values indicate the change in the diet score per one category increment in the socioeconomic status variables.

Figure 1 displays the mean diet scores categorized by different socioeconomic levels. A clear and significant trend was observed, showing that diet scores, indicative of healthier diets, increased with higher levels of education. Specifically, the mean diet score for individuals with higher education was 4.57 (95% CI: 4.56, 4.58), compared to 4.16 (95% CI: 4.15, 4.17) for those with lower levels of education. A similar pattern emerged with income levels: higher income individuals had a mean diet score of 4.55 (95% CI: 4.53, 4.57), while those with lower incomes scored 4.29 (95% CI: 4.28, 4.31). Furthermore, when assessing socioeconomic levels by area, the least deprived areas recorded an average score of 4.47 (95% CI: 4.45, 4.48). This score progressively decreased in more deprived areas, reaching 4.31 (95% CI: 4.30, 4.32) among participants from the most deprived areas.

**Figure 2:**
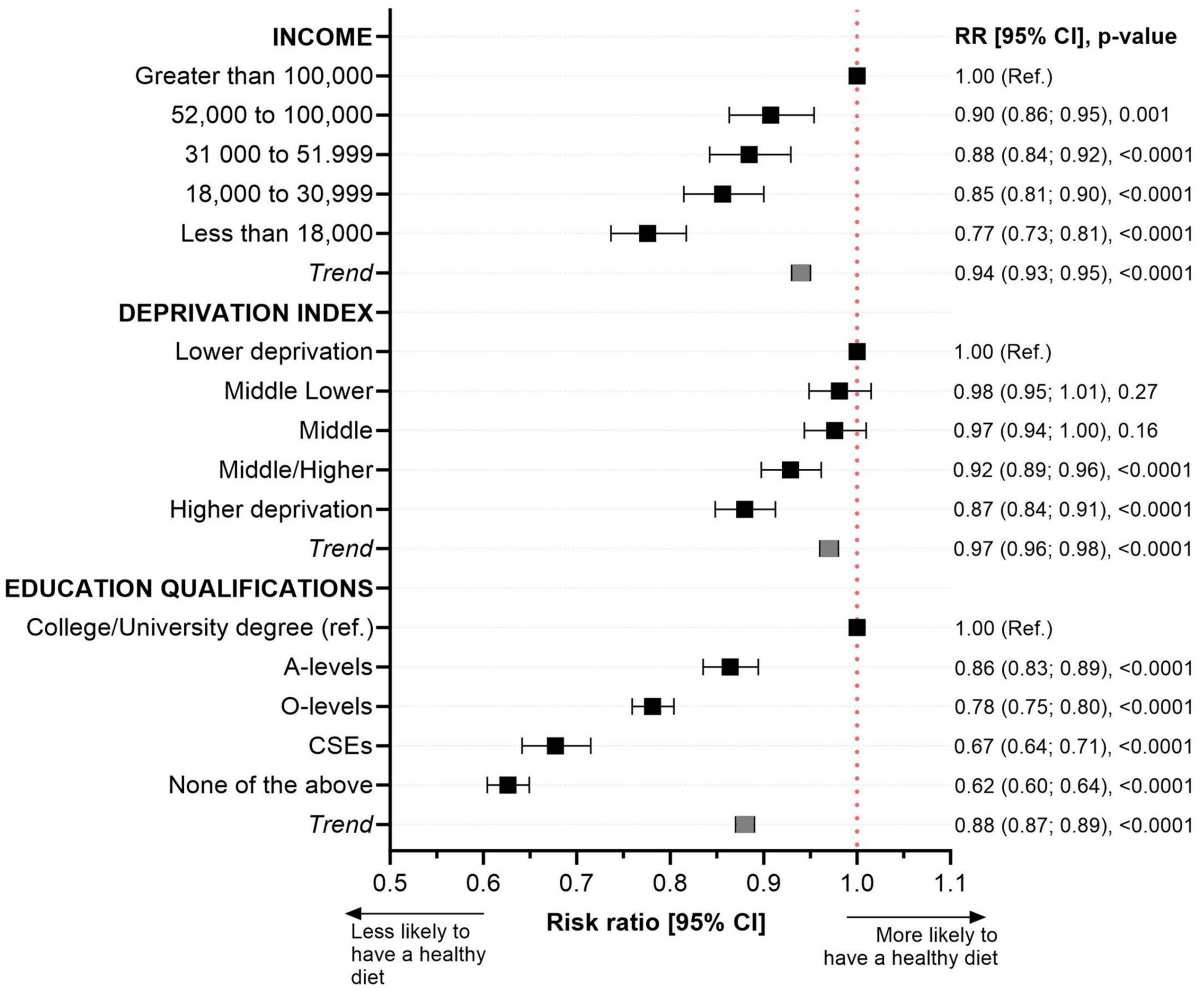
Likelihood of adhering to a healthy diet by income, deprivation, and education level. Data presented as risk ratio and their 95%CI. Adherence to a healthy diet quality score was defined as the top 25th percentile of the score (individuals with a score ≥ 7 points) while those in the lowest quartile with a score ≤ 3 points were classified as the least healthy diet. Analyses were adjusted for age, sex, ethnicity, smoking, multimorbidity, and body mass index. The reference group was those participants with the highest education or income levels or the least deprived. Trend values represent the change in risk ratio equivalent to one category increment in the exposure (education, income, and deprivation).

Figure 2 illustrates the likelihood of adhering to a healthy diet across various socioeconomic markers. When compared to individuals with the highest educational level, those with the lowest education were found to be 38% less likely to adhere to a healthy diet, as indicated by a relative risk (RR) of 0.62 (95% CI: 0.60; 0.64). Similarly, participants from the most deprived socioeconomic group had a 13% lower likelihood of adhering to a healthy diet (RR: 0.87, 95% CI: 0.84; 0.91). Furthermore, individuals with the lowest income were 23% less likely to maintain a healthy diet, with an RR of 0.77 (95% CI: 0.73; 0.81), as shown in Figure 2.

**Figure 3:**
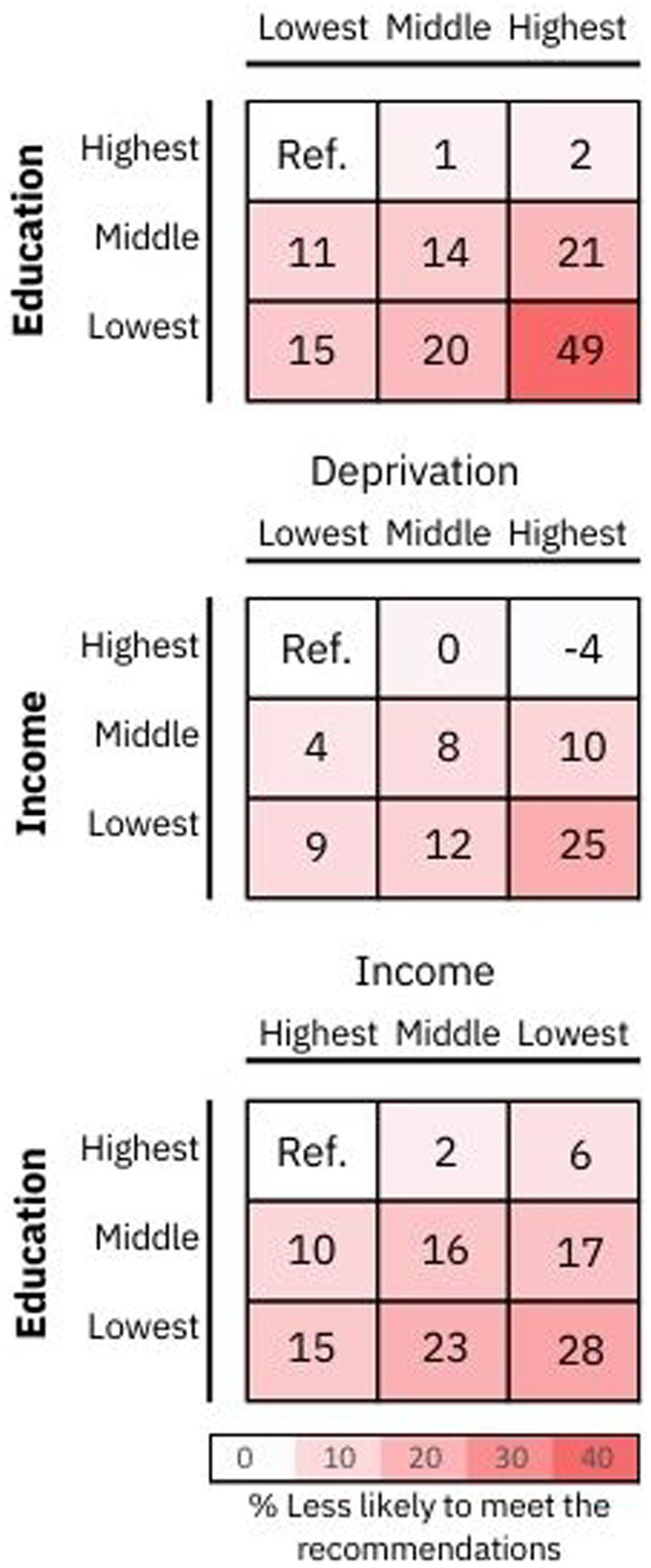
Adherence to a healthy diet by combined categories of socioeconomic status. Data presented the likelihood of meeting a healthy diet by combined socioeconomic status categories. Adherence to a healthy diet quality score was defined as the top 25th percentile of the score (individuals with a score ≥ 7 points), while those in the lowest quartile with a score ≤ 3 points were classified as the least healthy diet. A red color in the risk matrix represents a lower odd of meeting the healthy dietary recommendations. Risk ratios and 95% CI are presented in Supplementary Table S2.

Figure 3 displays adherence to a healthy diet across various combined socioeconomic categories. The analysis reveals that individuals with the least education, residing in the most deprived areas, were 49% less likely to adhere to a healthy diet compared to those with the highest educational level and living in the least deprived areas (RR: 0.51, 95% CI: 0.48; 0.55). Notably, high adherence to a healthy diet was observed consistently among individuals with a high educational level, regardless of their income level or whether they resided in affluent or deprived areas (refer to Figure 3 and Supplementary Table S2 for detailed results).

### 3.1 Individual food intake by markers of socioeconomic status

Supplementary Figure S1 illustrates how the consumption of individual food items varies with levels of deprivation. In the most deprived areas, as indicated by the deprivation index score, the consumption of red meat and cereals was significantly higher compared to more affluent areas. Additionally, the intake of processed meat and water was greater among individuals in the most deprived sectors. However, no notable differences were observed in the consumption of oily fish, fruit, and vegetables across different levels of area deprivation.

In contrast, individuals with higher education levels were found to consume more fruit and vegetables, oily fish, cereals, and water, and less processed meat. Interestingly, no clear association was observed between education level and red meat intake, as shown in Supplementary Figure S2. Moreover, similar consumption patterns, as noted with education levels, were also evident with regard to income levels, as detailed in Supplementary Figure S3.

## 4 Discussion

This study provides compelling evidence that non-adherence to a healthy diet and current dietary recommendations is more prevalent among individuals of lower SES. Notably, when combining various socioeconomic markers, we observed that adherence to dietary guidelines was consistently higher among those with higher educational levels, irrespective of their income or whether they resided in affluent or disadvantaged areas.

These findings align with those from a cross-sectional study in New Zealand, which indicated that adherence to a healthier “Mediterranean” dietary pattern correlated with higher education, while a “Western” dietary pattern was more common among those with lower education levels (1). This could be attributed to the fact that higher education often leads to greater awareness of the benefits of a healthy diet.

Furthermore, the least healthy diets in our study were characterized by inadequate consumption of cereals, water, fruits, and vegetables, and excessive intake of processed and red meats. This observation is consistent with prior evidence from cross-sectional studies (20). A systematic review in the UK, involving 1,491 participants, also reported better diet quality, particularly in fruit and vegetable intake and lower consumption of red and processed meats and oily fish, among more affluent individuals (21). When examining each food group independently, we noted suboptimal consumption of healthier foods and overconsumption of less healthy options among those with higher deprivation levels. Inverse relationships were evident between deprivation levels and spending on fruits and vegetables per person (22), which may be influenced by food environments and the accessibility and cost of foods of lower nutritional quality, echoing findings similar to ours (23).

However, this study is not without limitations. The UK Biobank’s participants are, on average, more affluent and healthier than the general UK population, which could affect the adherence estimates presented in our study. Additionally, the self-reported nature of the dietary questionnaire introduces a potential bias, as eating behaviors could be subject to misreporting.

Identifying these disparities in healthy diet adherence across different socioeconomic categories underscores crucial issues of equity in access to and affordability of nutritious foods. Our findings indicate that individuals with lower educational attainment, income, and those living in more deprived areas may encounter greater obstacles in adopting healthy dietary habits. These socioeconomic disparities could contribute to the broader health inequalities observed in the general population.

Future research should delve deeper into the mechanisms behind these associations and evaluate the effectiveness of interventions aimed at reducing disparities in healthy eating habits.

## 5 Conclusion

Our study’s findings highlight the critical need for targeted interventions aimed at improving adherence to healthy diets, especially among socioeconomically disadvantaged groups, with a particular focus on individuals with lower educational levels. These insights make a substantial contribution to existing research, underlining the importance of considering the interaction of various socioeconomic factors in the development of public health policies and initiatives. Furthermore, our results reveal the intricate nature of socioeconomic determinants of health, emphasizing the importance of integrating community-specific contexts into the framework of intervention strategies. This approach is vital to effectively address the nuanced challenges faced by different population segments in maintaining healthy dietary practices.

## Data availability statement

The data analyzed in this study is subject to the following licenses/restrictions: UK Biobank (https://www.ukbiobank.ac.uk). Requests to access these datasets should be directed to (https://www.ukbiobank.ac.uk).

## Ethics statement

The studies involving humans were approved by the UK Biobank study was approved by the North West Multi-Centre Research Ethics Committee (NHS National Research Ethics Service 16/NW/0274). Participants provided written informed consent for data collection, data analysis, and record linkage. This study is part of UK Biobank project 7155. The studies were conducted in accordance with the local legislation and institutional requirements. The participants provided their written informed consent to participate in this study.

## Author contributions

FC-M: Conceptualization, Formal analysis, Methodology, Validation, Writing – original draft, Writing – review & editing. SP-S: Writing – review & editing. JB: Writing – review & editing. NP: Writing – review & editing. AT: Validation, Writing – review & editing. FP-R: Conceptualization, Methodology, Writing – review & editing. JP: Writing – review & editing. FH: Writing – review & editing. NM-M: Writing – review & editing. CC-M: Conceptualization, Formal analysis, Methodology, Project administration, Supervision, Validation, Writing – original draft, Writing – review & editing, Visualization. RM-L: Methodology, Project administration, Supervision, Writing – review & editing. GM-R: Methodology, Project administration, Supervision, Writing – review & editing.

## Footnotes

^1^ https://www.ukbiobank.ac.uk

## References

[1] Afshin A Sur PJ Fay KA Cornaby L Ferrara G Salama JS. Health effects of dietary risks in 195 countries, 1990–2017: a systematic analysis for the global burden of disease study 2017. Lancet. (2019) 393:1958–72. doi: 10.1016/S0140-6736(19)30041-8, PMID: 30954305 PMC6899507

[2] Wang P Song M Eliassen AH Wang M Fung TT Clinton SK. Optimal dietary patterns for prevention of chronic disease. Nat Med. (2023) 29:719–28. doi: 10.1038/s41591-023-02235-5, PMID: 36914892 PMC10294543

[3] Collaborators GBDRF. Global, regional, and national comparative risk assessment of 84 behavioural, environmental and occupational, and metabolic risks or clusters of risks for 195 countries and territories, 1990-2017: a systematic analysis for the global burden of disease study 2017. Lancet. (2018) 392:1923–94. doi: 10.1016/S0140-6736(18)32225-6 30496105 PMC6227755

[4] GBD 2019 Risk Factors Collaborators. Global burden of 87 risk factors in 204 countries and territories, 1990-2019: a systematic analysis for the global burden of disease study 2019. Lancet. (2020) 396:1223–49. doi: 10.1016/S0140-6736(20)30752-2, PMID: 33069327 PMC7566194

[5] Drewnowski A Specter S. Poverty and obesity: the role of energy density and energy costs. Am J Clin Nutr. (2004) 79:6–16. doi: 10.1093/ajcn/79.1.6, PMID: 14684391

[6] Petermann-Rocha F Ho FK Foster H Boopor J Parra-Soto S Gray SR. Nonlinear associations between cumulative dietary risk factors and cardiovascular diseases, Cancer, and all-cause mortality: a prospective cohort study from UK biobank. Mayo Clin Proc. (2021) 96:2418–31. doi: 10.1016/j.mayocp.2021.01.036, PMID: 34366141

[7] Stefler D Malyutina S Kubinova R Pajak A Peasey A Pikhart H. Mediterranean diet score and total and cardiovascular mortality in Eastern Europe: the HAPIEE study. Eur J Nutr. (2017) 56:421–9. doi: 10.1007/s00394-015-1092-x, PMID: 26578528 PMC5290049

[8] Bonaccio M Di Castelnuovo A Pounis G Costanzo S Persichillo M Cerletti C. High adherence to the Mediterranean diet is associated with cardiovascular protection in higher but not in lower socioeconomic groups: prospective findings from the Moli-sani study. Int J Epidemiol. (2017) 46:1478–87. doi: 10.1093/ije/dyx145, PMID: 29040542

[9] Lakshman R McConville A How S Flowers J Wareham N Cosford P. Association between area-level socioeconomic deprivation and a cluster of behavioural risk factors: cross-sectional, population-based study. J Public Health. (2011) 33:234–45. doi: 10.1093/pubmed/fdq072, PMID: 20884643 PMC3714999

[10] Mayén A-L Marques-Vidal P Paccaud F Bovet P Stringhini S. Socioeconomic determinants of dietary patterns in low-and middle-income countries: a systematic review. Am J Clin Nutr. (2014) 100:1520–31. doi: 10.3945/ajcn.114.089029, PMID: 25411287

[11] Miller V Yusuf S Chow CK Dehghan M Corsi DJ Lock K. Availability, affordability, and consumption of fruits and vegetables in 18 countries across income levels: findings from the prospective urban rural epidemiology (PURE) study. Lancet Glob Health. (2016) 4:e695–703. doi: 10.1016/S2214-109X(16)30186-3, PMID: 27567348

[12] Aggarwal A Monsivais P Cook AJ Drewnowski A. Does diet cost mediate the relation between socioeconomic position and diet quality? Eur J Clin Nutr. (2011) 65:1059–66. doi: 10.1038/ejcn.2011.72, PMID: 21559042 PMC3157585

[13] Sudlow C Gallacher J Allen N Beral V Burton P Danesh J. UK biobank: an open access resource for identifying the causes of a wide range of complex diseases of middle and old age. PLoS Med. (2015) 12:e1001779. doi: 10.1371/journal.pmed.1001779, PMID: 25826379 PMC4380465

[14] Townsend P Phillimore P Beattie A. Health and deprivation: inequality and the. London: Croom Helm (1988).

[15] Hagenaars SP Gale CR Deary IJ Harris SE. Cognitive ability and physical health: a Mendelian randomization study. Sci Rep. (2017) 7:2651. doi: 10.1038/s41598-017-02837-3, PMID: 28572633 PMC5453939

[16] Department of Health. (2016). The eatwell plate. Available at:https://www.forumhealthcentre.nhs.uk/your-health/the-eatwell-plate

[17] EFSA NDA Panel. Scientific opinion on establishing food‐based dietary guidelines. EFSA J. (2010) 8:8. doi: 10.2903/j.efsa.2010.1460

[18] Waijers PMCM Feskens EJM Ocké MC. A critical review of predefined diet quality scores. Br J Nutr. (2007) 97:219–31. doi: 10.1017/S0007114507250421, PMID: 17298689

[19] Obesity WHOCo, World Health O. Obesity: Preventing and managing the global epidemic: Report of a WHO consultation. Geneva: World Health Organization (2000). 11234459

[20] Darmon N Drewnowski A. Does social class predict diet quality? Am J Clin Nutr. (2008) 87:1107–17. doi: 10.1093/ajcn/87.5.1107, PMID: 18469226

[21] Maguire ER Monsivais P. Socio-economic dietary inequalities in UK adults: an updated picture of key food groups and nutrients from national surveillance data. Br J Nutr. (2015) 113:181–9. doi: 10.1017/S0007114514002621, PMID: 25399952 PMC4351901

[22] Whybrow S Hollis JL Macdiarmid JI. Social deprivation is associated with poorer adherence to healthy eating dietary goals: analysis of household food purchases. J Public Health. (2017) 40:e8–e15. doi: 10.1093/pubmed/fdx007 28158783

[23] Morland K Wing S Diez RA. The contextual effect of the local food environment on residents' diets: the atherosclerosis risk in communities study. Am J Public Health. (2002) 92:1761–8. doi: 10.2105/AJPH.92.11.1761, PMID: 12406805 PMC1447325

