# Supplementary material for: Adherence to dietary recommendations by socioeconomic status in the United Kingdom biobank cohort study
Source: Front Nutr. 2024 May 1;11:1349538. doi: 10.3389/fnut.2024.1349538 (PMC11094746; doi:10.3389/fnut.2024.1349538)
Supplement: Supplementary file 1 [file Data_Sheet_1.docx]

Supplementary Material

# Supplementary Data

**Adherence to dietary recommendations by individual and are-based socioeconomic status in the UK Biobank cohort study.**

All food items were dichotomised into meeting and not meeting dietary recommendations using cut-offs derived from the UK and European food-based dietary intake guidelines where these existed (the Eatwell guide and the Food-Based Dietary Guidelines from the European Food Safety Authority). For those food items without a specific recommendation of intake, the median was used [1-3]. More details are provided in Supplementary Table 1.

# Supplementary Figures and Tables

**Supplementary Table S1. Foods groups and recommendations used to derive the healthy diet score.**

| **Food item** | **Categories reported from the touch-screen questionnaire** | **Categorization** |
| --- | --- | --- |
| Fruit & vegetables (regrouped from fruit, dried fruit & Vegetable) | Serving/day | ≥5 serving/day (Ref.) <5 serving/day |
| Total fish intake (regrouped from Both total non- oily fish and oily fish) | Never Less than once a week Once a week 2-4 times a week 5-6 times a week Once or more daily | ≥2 times a week (at least once a week of each category) (Ref.)  < once a week of each one |
| Processed meat intake | Never Less than once a week Once a week 2-4 times a week 5-6 times a week Once or more daily | ≤Once a week (Ref.)  > Once a week |
| Red meat (regrouped from beef, pork and lamb) | Never Less than once a week Once a week 2-4 times a week 5-6 times a week Once or more daily | >Once a week  ≤Once a week (Ref.) |
| Milk type used | Full cream Semi-skimmed Skimmed Soya another type of milk Never rarely have milk | Semi- skimmed/skimmed (Ref.) Full cream/ another type of milk/ never rarely have milk |
| Spread type | Never/rarely Butter Other type/ margarine Flora pro-active/benecol | Never/rarely (Ref.) Another selection |
| Cereal intake | Bowls/week | >5 bowls (Ref.) ≤5 bowls |
| Salt added to food | Never/rarely, Sometimes, Usually, Always | Never/rarely (Ref.) Another selection |
| Water intake | Glasses/day | ≥6 glasses (Ref.) <6 glasses |

**This table is an updated version from Petermann-Rocha, F., et al. (2021) [4].*


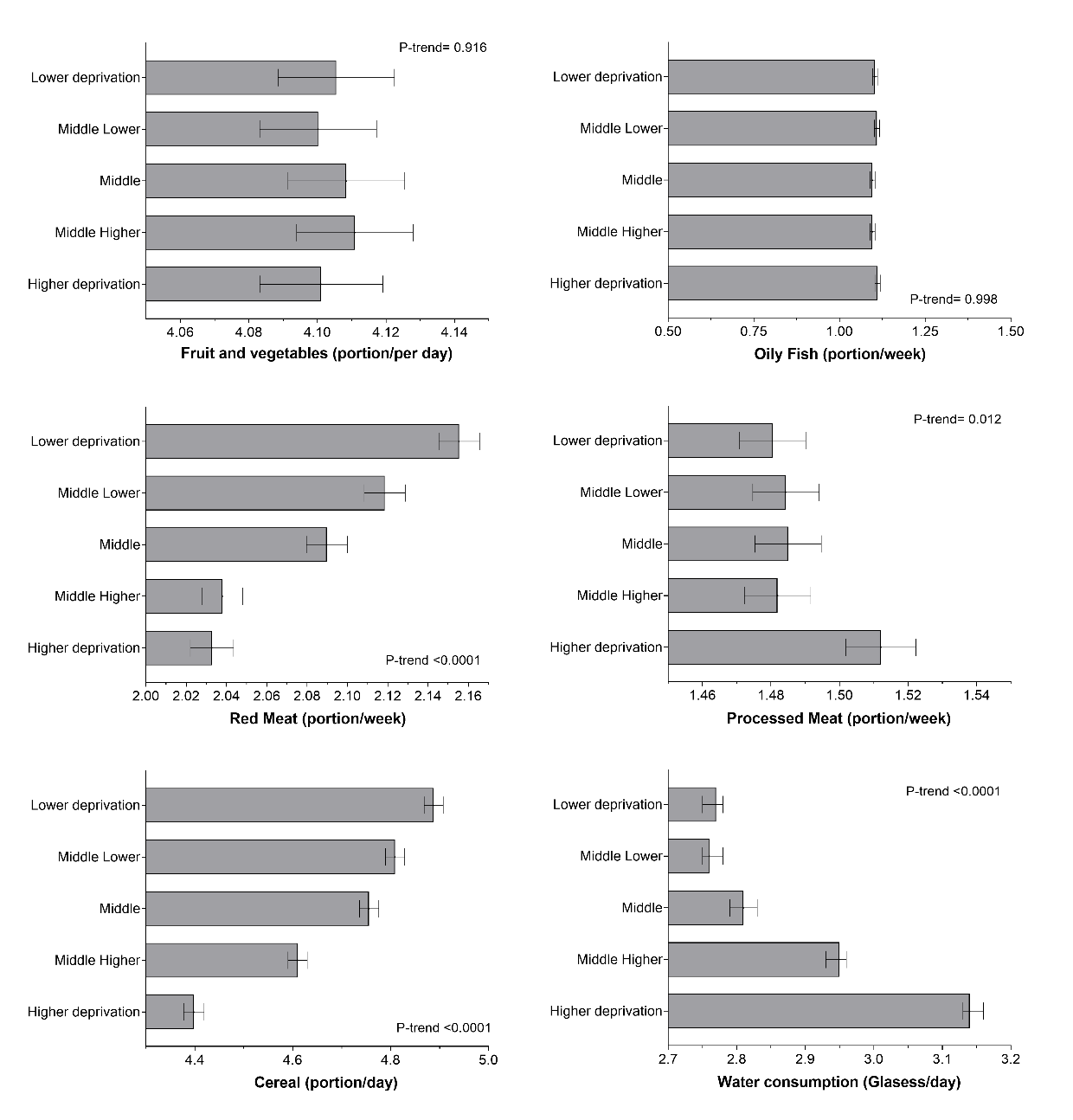


**Supplementary Figure S1. Mean intake of dietary recommendations by categories of deprivation.**

The data are presented as adjusted mean intake and 95%CI. Analyses were adjusted for age, sex, ethnicity, smoking, multimorbidity and body mass index. Trend p-values were estimated using linear regression analysis.


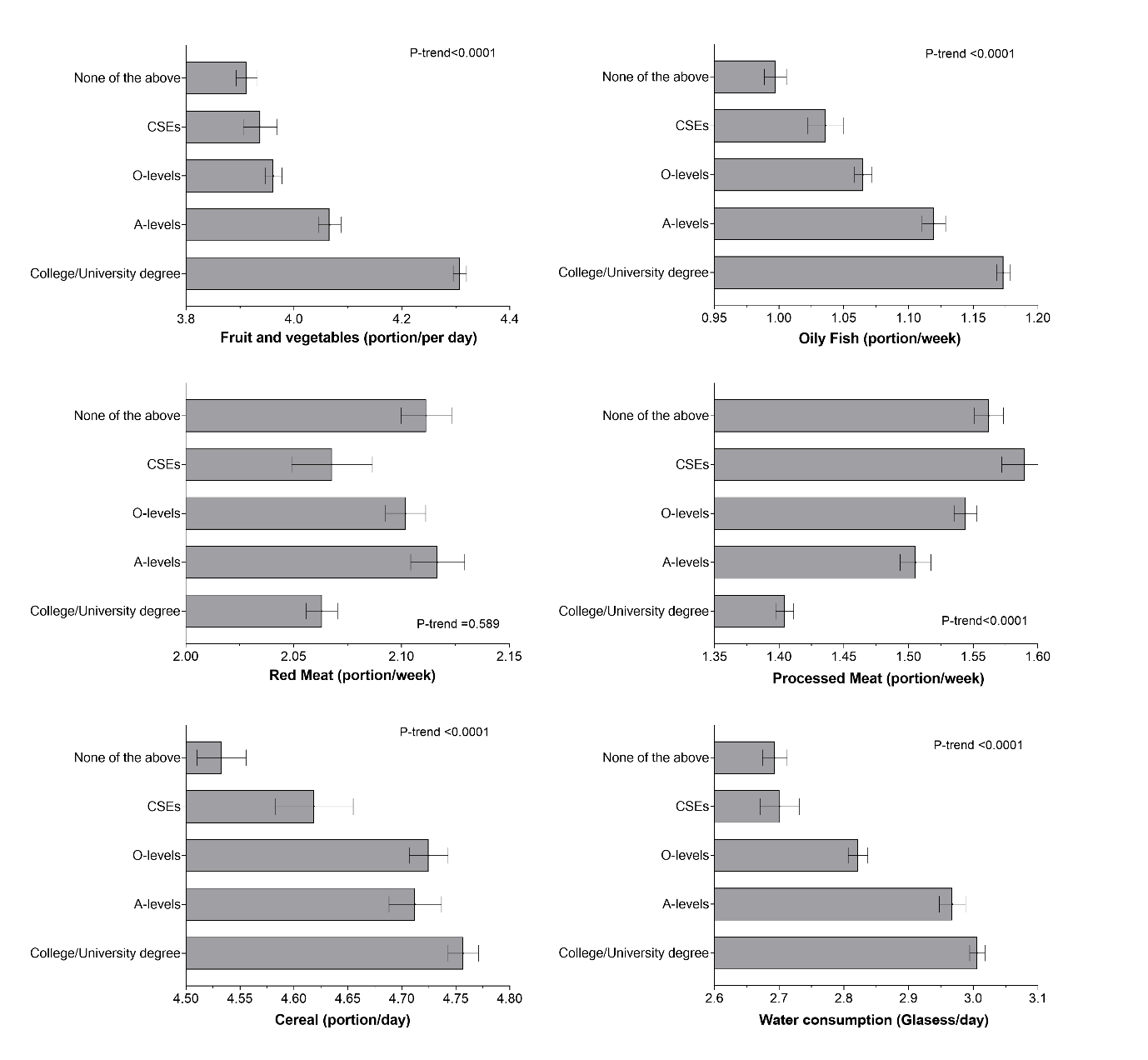


**Supplementary Figure S2. Mean intake of dietary recommendations by categories of education.**

The data are presented as adjusted mean intake and 95%CI. Analyses were adjusted for age, sex, ethnicity, smoking, multimorbidity and body mass index. Trend p-values were estimated using linear regression analysis.


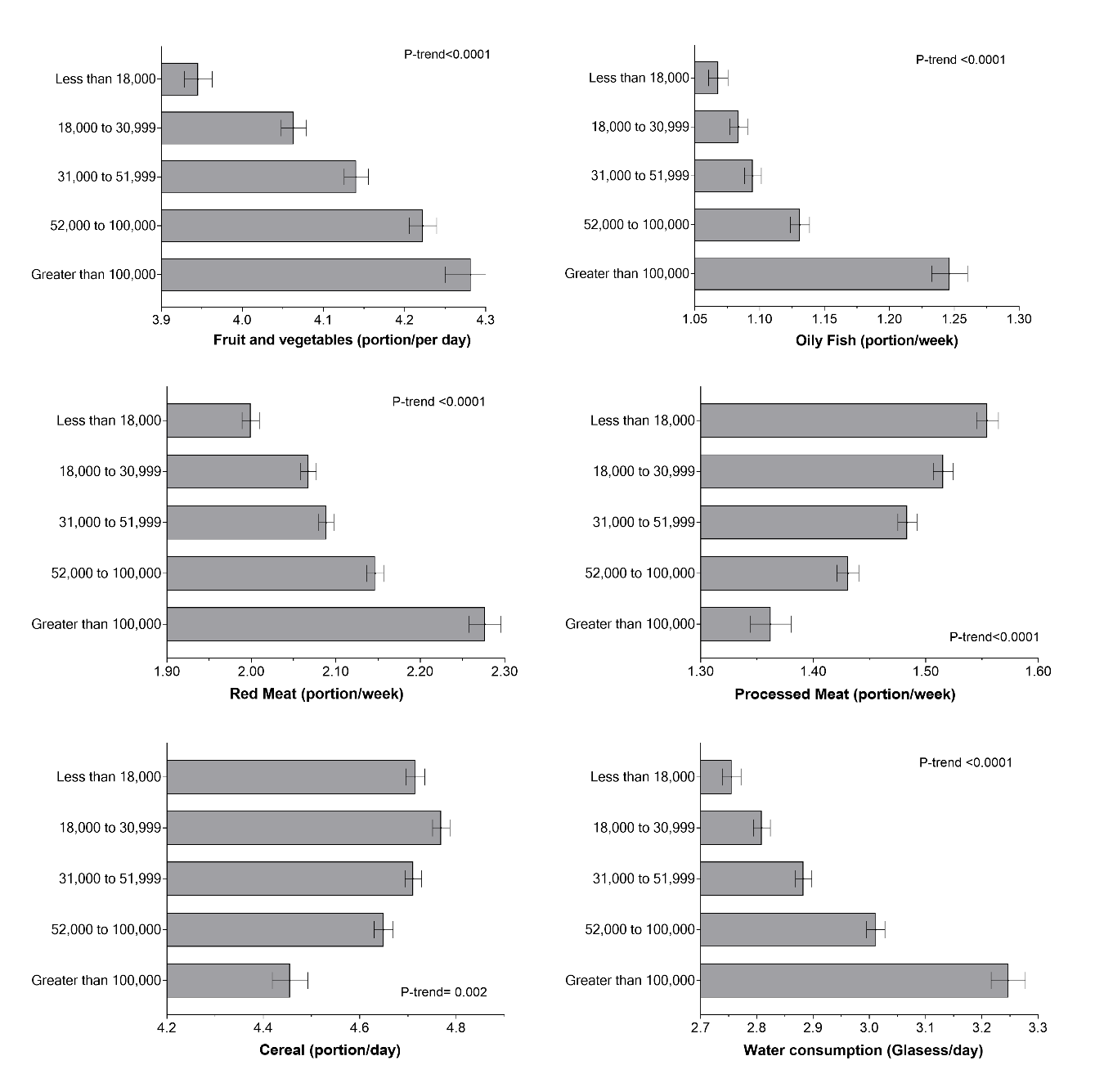


**Supplementary Figure S3. Mean intake of dietary recommendations by categories of income.**

The data are presented as adjusted mean intake and 95%CI. Analyses were adjusted for age, sex, ethnicity, smoking, multimorbidity and body mass index. Trend p-values were estimated using linear regression analysis.

**Supplementary Table S2**. Risk ratio for adherence to healthy eating by combined categories of socioeconomic status.

|  | **Deprivation** | | |
| --- | --- | --- | --- |
| **Education** | Lowest (least deprived) | Middle | Highest (most deprived) |
| Highest | 1.00 (Ref.) | 1.01 (0.96; 1.06), p=0.702 | 1.02 (0.97; 1.08), p=0.364 |
| Middle | 0.82 (0.77; 0.87), p<0.0001 | 0.78 (0.73; 0.83), p<0.0001 | 0.74 (0.69; 0.79), p<0.0001 |
| Lowest | 0.73 (0.67; 0.79), p<0.0001 | 0.70 (0.65; 0.75), p<0.0001 | 0.51 (0.48; 0.55), p<0.0001 |
|  | **Deprivation** | | |
| **Income** | Lowest (least deprived) | Lowest (least deprived) | Lowest (least deprived) |
| Highest | 1.00 (Ref.) | 1.01 (0.88; 1.15), p=0.880 | 1.12 (0.98; 1.28), p=0.092 |
| Middle | 0.94 (0.85; 1.03), p=0.230 | 0.91 (0.82; 1.00), p=0.056 | 0.87 (0.78; 0.96), p=0.010 |
| Lowest | 0.89 (0.80; 1.00), p=0.052 | 0.86 (0.77; 0.95), p=0.005 | 0.68 (0.62; 0.75) p<0.0001 |
|  | **Income** | | |
| **Education** | **Highest** | **Middle** | **Lowest** |
| Highest | 1.00 (Ref.) | 0.99 (0.93; 1.05), p=0.773 | 0.97 (0.91; 1.05), p=0.581 |
| Middle | 0.84 (0.72; 0.99), p=0.046 | 0.73 (0.68; 0.78), p<0.0001 | 0.78 (0.73; 0.84), p<0.0001 |
| Lowest | 0.83 (0.60; 1.13), p=0.249 | 0.61 (0.55; 0.68), p<0.0001 | 0.60 (0.56; 0.64), p<0.0001 |

Data presented as risk ratio and their 95% CI. A risk ratio below 1 indicates a lower adherence to a healthy diet (defined as a score of ≤3 points), while a risk ratio above 1 suggests a higher adherence to a healthy diet (defined as a score ≥7 points). The reference groups were defined as those with the most affluent category for each of the socioeconomic status variables used (i.e., high education and lowest deprivation). Analyses were adjusted for age, sex, ethnicity, smoking, multimorbidity and body mass index.

# References Supplementary

1. Buttriss, J.L. (2016), The Eatwell Guide refreshed. Nutr Bull, 41: 135 -141. https://doi.org/10.1111/nbu.12211
2. Department, of, Health. The Eatwell Plate. 2016.
3. EFSA Panel on Dietetic Products N, Allergies. Scientific Opinion on establishing Food-Based.
4. Petermann-Rocha, F.; Ho, F.K.; Foster, H.; Boopor, J.; Parra-Soto, S.; Gray, S.R.; Mathers, J.C.; Celis-Morales, C.; Pell, J.P. Nonlinear Associations Between Cumulative Dietary Risk Factors and Cardiovascular Diseases, Cancer, and All-Cause Mortality: A Prospective Cohort Study From UK Biobank. Mayo Clinic Proceedings 2021, 96, 2418-2431, doi:https://doi.org/10.1016/j.mayocp.2021.01.036.
